# IMP1 regulates UCA1-mediated cell invasion through facilitating UCA1 decay and decreasing the sponge effect of UCA1 for miR-122-5p

**DOI:** 10.1186/s13058-018-0959-1

**Published:** 2018-04-18

**Authors:** Yanchun Zhou, Xiuhua Meng, Shaoying Chen, Wei Li, Delin Li, Robert Singer, Wei Gu

**Affiliations:** 1 Department of Pathophysiology, The Key Immunopathology Laboratory of Guangdong Province, Shantou University Medical College, Shantou, 515031 Guangdong Province China; 2 Department of Anatomy and Structural Biology, Albert Einstein College of Medicine, Bronx, NY 10461 USA

**Keywords:** lncRNA, RNA-binding protein, IMP1, UCA1, IMP1-UCA1 interaction, UCA1-miR122-5p interaction

## Abstract

**Background:**

Long noncoding RNAs (LncRNAs) represent a class of widespread and diverse endogenous RNAs that can posttranscriptionally regulate gene expression through the interaction with RNA-binding proteins and micro RNAs (miRNAs). Here, we report that in breast carcinoma cells, the insulin-like growth factor 2 messenger RNA binding protein (IMP1) binds to lncRNA urethral carcinoma-associated 1 (UCA1) and suppresses the UCA1-induced invasive phenotype.

**Methods:**

RT-qPCR and RNA sequence assays were used to investigate the expression of UCA1 and miRNAs in breast cancer cells in response to IMP1 expression. The role of IMP1-UCA1 interaction in cell invasion was demonstrated by transwell analysis through loss-of-function and gain-of-function effects. RNA pull-down and RNA binding protein immunoprecipitation (RIP) were performed to confirm the molecular interactions of IMP1-UCA1 and UCA1-miR-122-5p involved in breast cancer cells.

**Results:**

In breast cancer cells, IMP1 interacts with UCA1 via the “ACACCC” motifs within UCA1 and destabilizes UCA1 through the recruitment of CCR4-NOT1 deadenylase complex. Meanwhile, binding of IMP1 prevents the association of miR-122-5p with UCA1, thereby shifting the availability of miR-122-5p from UCA1 to the target mRNAs and reducing the UCA1-mediated cell invasion. Accordingly, either IMP1 silencing or UCA1 overexpression resulted in reduced levels of free miR-122-5p within the cytoplasm, affecting miR-122-5p in regulating its target mRNAs.

**Conclusions:**

Our study provides initial evidence that interaction between IMP1 and UCA1 enhances UCA1 decay and competes for miR-122-5p binding, leading to the liberation of miR-122-5p activity and the reduction of cell invasiveness.

**Electronic supplementary material:**

The online version of this article (10.1186/s13058-018-0959-1) contains supplementary material, which is available to authorized users.

## Background

With numerous non-coding RNA transcripts (ncRNA) being identified over recent years, investigation of the biological functions of ncRNAs has become an attractive research area [1–4]. To date, since the effects of small ncRNAs such as microRNAs have been widely studied [5, 6], attention has been shifted towards the impact of long non-coding RNA (lncRNA) on pathologic diseases, including autoimmune disease, neurological disorders and cancer [7, 8].

LncRNAs have recently been considered as master regulators that modulate gene expression through transcriptional and posttranscriptional mechanisms. For example, Hox transcript antisense RNA (HOTAIR) plays a critical role in regulating the chromatin state through interaction with the polycomb repressive complex 2 [9]. In human fibroblasts, association between promoter of CDKN1A antisense DNA damage activated RNA (PANDA) and transcription factor nuclear transcription factor Y alpha (NF-YA) limits the expression of pro-apoptotic genes [10]. LncRNAs also regulate numerous posttranscriptional processes, including translation and mRNA decay as exemplified by beta secretase (BACE)-AS, an endogenous antisense transcript of BACE1 that base-pairs BACE1 mRNA to enhance the stability of BACE1 mRNA, thereby increasing the levels of BACE1 protein translation [11].

Recently, a large number of lncRNAs have been reported to function as competing endogenous RNA (ceRNA). These lncRNAs can serve as molecular sponges for miRNAs, and hence functionally affect the activity of other RNA transcripts targeted by the sponged miRNAs [12–14]. Human urothelial carcinoma associated 1 (UCA1) belongs to this type of regulatory lncRNA. UCA1 is highly expressed in breast, gastric and colorectal cancers, indicating a common important role in human cancers [15, 16]. UCA1 contributes to the progression of carcinoma cells by interacting with miRNAs including miR-216b, miR-143 and miR-204-5p [16–18]. Since these miRNAs negatively regulate the posttranscriptional expression of genes that are involved in many fundamental cell processes, such as proliferation, differentiation and invasion, the sponge effect of UCA1 on the miRNAs pinpoints its biological importance in the regulation of gene expression.

It has been recognized that one of the mechanisms by which lncRNAs regulate posttranscriptional gene expression is through the interaction with RNA-binding proteins (RBPs) [19–21]. In the same way, RBPs also bind to a large and heterogeneous class of functional lncRNAs to modulate their biological effects [22]. Protein–RNA interactions are important aspects of many biological processes that go beyond the already established steps of mRNA production, e.g. transcription, splicing, decay and translation [7, 23–25]. In tumor cells, many RBPs, such as DNA methyltransferases (DNMTs), heterochromatin protein 1, polycomb-group and trithorax-group proteins, are able to bind lncRNAs and exert their biological functions [26–28]. Recently, a study reported that in human liver cancer cells, insulin-like growth factor 2 mRNA binding protein (IGF2BP), also known as IMP1 specifically interacts with the lncRNA highly upregulated in liver cancer (HULC) and promotes the decay of HULC through Ccr4-Not 1 (CNOT1)-mediated deadenylation [29].

As an RNA-binding protein, IMP1 has been implicated in many aspects of RNA regulation [30]. In a variety of cell types, IMP1 mediates the localized translation of β-actin mRNA at the leading edge, through which to enhance cell polarity [31, 32]. Interaction of IMP1 to β-actin mRNA requires the KH34 domains of IMP1, which recognizes a specific “ACACCC” motif within the 3’ untranslated region (UTR) of β-actin mRNA [33, 34]. In breast cancer cells, loss of IMP1 function deregulates mRNAs normally associated with the protein, resulting in decreased cell polarity and increased invasive ability [35, 36]. Based on the fact that IMP1 could also regulate lncRNA HULC expression in liver cancer cells [29], investigation of IMP1-lncRNA interactions could uncover new pathways for IMP1-mediated biological processes.

In the present study, we show that in breast cancer cells IMP1 binds to the ACACCC motifs of lncRNA UCA1 via the KH34 domain of the protein. Interaction between IMP1 and UCA1 destabilizes UCA1 and suppresses the UCA1-induced invasive phenotype. We demonstrated that UCA1 acts as a sponge for endogenous miR-122-5p, a repressor of several mRNAs related to cell invasion. Binding of IMP1 to UCA1 not only destabilizes UCA1, but also prevents the association between UCA1 and miR-122-5p, therefore shifting the miRNA availability from UCA1 to target mRNAs. Accordingly, either IMP1 silencing or UCA1 overexpression reduces the ability of miR-122-5p to regulate target mRNAs. Our study provides initial evidence that interaction between IMP1 and UCA1 increases UCA1 decay and reduces the sponge effect of UCA1 to miRNAs to eventually decrease the oncogenic role of UCA1.

## Methods

### Reagents

Sources of primary antibodies were IMP1 and glyceraldehyde-3-phosphate dehydrogenase (GAPDH) from Cell Signaling (Danvers, USA); CNOT1 and Ago2 from Santa Cruz Biotechnology (Dallas, USA); green fluorescent protein (GFP) and Flag from Sigma Aldrich (USA). Secondary antibodies conjugated with horseradish peroxidase (HRP) were purchased from Santa Cruz Biotechnology. UCA1 short interfering RNAs (siRNAs), IMP1-siRNA and control siRNA were purchased from Thermo Fisher Scientific (USA). Polymerase chain reaction (PCR) primers used in the study were ordered from Tiangen Biotech Co. (Beijing, China) and are listed in Additional file 1: Table S1.

### Cell lines and culture conditions

The human embryonic kidney 293 T cell line, human breast cancer cell lines MDA-MB-231 (MDA231), T47D and MCF7 were obtained from American Type Culture Collection (ATCC, Manassas, VA, USA). IMP1 knockdown T47D cells, MDA231/IMP1-GFP and MDA231/GFP cell lines were previously generated [36, 37]. UCA1 overexpression and knockdown MDA231 and T47D stable cell lines were established by stable infection of lentivirus expressing UCA1 or UCA1-short hairpin RNA (shRNA). Cells were cultured in Dulbecco’s modified Eagle’s medium (DMEM) (Thermo Fisher Scientific, USA) supplemented with 10% fetal bovine serum (FBS), 100 U/ml penicillin, and 100 μg/ml streptomycin at 37 °C in a humid environment with 5% CO_2_.

### Plasmid construction

Human UCA1 (1.4 kb, gene ID: EU334869) complementary DNA (cDNA) fragment was amplified by RT-PCR from total RNA extracted from MDA231 cells using the primers listed in Additional file 1: Table S1. Amplified UCA1 was cloned into the pCIP2 lentivirus plasmid at the Not I and Bam HI sites. pCIP2-UCA1-MS2_6_ was constructed by introducing a six-repeat MS2 hairpin structure into the BamHI site of the pCIP2-UCA1 plasmid. Mutations of the IMP1 binding motifs and the miR-122-5p binding site within the UCA1 sequence were performed using a Q5® Site-Directed Mutagenesis Kit (NEB, USA). PsiCHECK 2 (Promega) was used for luciferase assays (p-luc) and reporter construction. To construct luciferase reporter genes, the DNA fragments of wild-type (WT)-UCA1 and mutant UCA1 were cloned to the 3’ Renilla luciferase gene of the PsiCHECK 2 plasmid (denoted as pluc-UCA1 and pluc-mUCA1). All constructs were verified by sequencing analysis.

### Cell transfection, lentivirus assembly and infection

Transfection of miRNA mimics, siRNAs, oligonucleotides and plasmids was conducted using Lipofectamine™ 2000 transfection reagent (Invitrogen, USA) following the protocol recommended by the manufacturer. At 48 h after transfection, cells were collected and used for further investigations. Lentivirus was generated by co-transfecting 293 T cells with the lentiviral vector and packaging plasmids [38]. Supernatants were collected 48 h later, filtered through 0.45-μm filters and concentrated using Lenti-X concentrator (Clontech). Concentrated virus was used to infect cells immediately or stored at − 80 °C for later usage. Stably infected cell lines were selected with 2–5 μg/ml puromycin for about 2 weeks. The expression levels of expressed genes were detected by RT-qPCR.

### Isolation of IMP1 RNP complexes and RNA extraction

Briefly, cells at 80–90% confluence were scraped from culture dishes and washed twice in ice-cold phosphate-buffered saline (PBS). Cells were lysed in an ice-cold lysis buffer (20 mM Tris·HCl, pH 7.5, 50 mM NaCl, 5 mM MgCl_2_, 0.5% NP-40) containing protease inhibitors (Roche Applied Science) and 200 U/ml RNasin (Takara, China). Lysates were centrifuged at 12,000 rpm at 4 °C for 15 min to remove cell debris. Supernatants were incubated with antibodies conjugated to protein A agarose beads (Thermo Fisher Scientific) at 4 °C for 4 h with gentle rotation. After incubation, the supernatant was removed by brief centrifugation and the beads were washed extensively using lysis buffer, followed by adding 0.5 ml of TRIzol. RNAs associated with IMP1 were extracted and analyzed by RT-qPCR.

### LncRNA microarray

Total RNAs from MDA231/GFP and MDA231/IMP1-GFP cells were extracted and used for lncRNA microarray analysis at the Shanghai Biotechnology Corporation (China). The raw microarray data have been deposited in Gene Expression Omnibus (GEO) database [GEO:GSE91057].

### MS2 pulldown analysis and miRNA sequencing

MBP-MCP pulldown assays were performed as previously described [38]. Briefly, amylose beads (NEB, USA) were incubated with purified MBP-MCP for 1 h at 4 °C. Cell lysates prepared from cultured UCA1-MS2 or mutant UCA1-MS2 cells were incubated with MBP-BCP coated amylose resin at 4 °C in the presence of RNase and protease inhibitors. After incubation for 5 h and extensive washing, bound UCA1-MS2 RNP complexes were eluted with 100 μl lysis buffer containing 20 mM maltose. Aliquots of the eluted materials were used for analyzing UCA1-associated protein(s) by western blot, and the rest were used for RNA extraction with TRIzol reagent (Invitrogen) and measured for enrichment of the RNAs by RT-qPCR experiments. Where indicated, miRNAs associated with UCA1 in the precipitates were extracted and analyzed by miRNA sequencing at the Guangzhou RiboBio Co. (China). The miRNA sequencing data have been deposited in the GEO database [GEO:GSE62638].

### Reverse transcription and real-time PCR (quantitative (q)PCR)

Total RNA from cultured cells was prepared using an RNA extraction kit (Tiangen, China). First-strand cDNA was synthesized using the PrimeScript RT reagent kit with gDNA Eraser (Takara, China). qPCR was performed using a QuantiTet SYBR Green PCR kit and measured in an ABI Prism 7500 (Applied Biosystems, Foster City, CA, USA). qRCR for miRNAs was performed using a miRcute miRNA Isolation kit (Tiangen, China). Briefly, total RNAs were extracted and miRNAs were polyadenylated and reverse transcribed using the universal reverse PCR primer provided in the kit. The resulting cDNA was then subjected to qPCR. U6 small nuclear RNA (snRNA) was used as an internal control. The specific forward primers for miR-122-5p, miR-185-5p, miR-10b-5p, and control U6 are listed in Additional file 1: Table S1. Each sample was analyzed in triplicate. The 2^-ΔΔct^ method was used to calculate the relative gene expression levels.

### Western blot analysis

Total cell lysates were prepared as previously described [35]. Protein samples were separated by 4–12% SDS-PAGE (Invitrogen Inc., USA) and transferred onto 0.45-μm nitrocellulose membranes. Following 1-h incubation in 5% fat-free milk, the membranes were probed with selected primary antibodies overnight at 4 °C. Blots were then washed, incubated for 1 h with respective secondary antibodies, and visualized using enhanced chemiluminescence reagents (Amersham Inc., USA). For sequential immunoblotting experiments, the blots were washed with Tris-buffered saline, treated with Restore Western Blot Stripping Buffer (Thermo Fisher Scientific, USA), washed and re-blocked, and incubated with primary antibodies.

### Cell invasion assays

Cell invasion assays were performed using Matrigel® Invasion Chambers (8-μm pore size, Corning Costar Corporation) according to the manufacturer’s protocol. Cells (2 × 10^4^) were suspended in 200 μl DMEM containing 1% FBS and added to the upper chamber. DMEM (700 μl) containing 10% FBS was added to the lower chamber. Cells were incubated for 20 h at 37 °C with 5% CO^2^. After incubation, cells on the upper surface of the membrane were removed with a cotton swab. Cells on the lower surface of the membrane were fixed with 4% paraformaldehyde for 15 min and stained with 0.2% crystal violet for 10 min. The numbers of invasive cells were calculated on the entire lower surface. The experiment was repeated three times in triplicate.

### Cell proliferation assay

Cell proliferation was determined by a 3-(4, 5-dimethylthiazolyl-2-yl)-2–5 diphenyltetrazolium bromide (MTT) assay. Cells were plated in 96-well plates at 5 × 10^3^ cells per well in a final volume of 100 μl. After incubation for 2, 24, 48 and 72 h, 10 μl (5 mg/ml) of MTT (Sigma, USA) solution was added to each well. After 4 h of incubation at 37 °C, the supernatant was removed and 150 μl of dimethyl sulfoxide (DMSO) was added. Absorbance at 570 nm was measured by a microplate spectrophotometer (BioRad, USA). Each experiment was performed in triplicate, each involving five replicates.

### Bioinformatics methods

Potential microRNA binding sites of UCA1 were predicted by algorithms obtained from the Segal Laboratory (http://132.77.150.113/pubs/mir07/mir07_prediction.html), by RegRNA (http://regrna.mbc.nctu.edu.tw/html/prediction.html) and by online microRNA software (http://www.microRNA.org). Putative miR-122-5p and miR-185-5p target genes were predicted using a miRNA target prediction algorithm Target Scan (http://www.targetscan.org/).

### Statistical analysis

For evaluation of RNA levels, RT-PCR products were first confirmed with agarose electrophoresis. For statistical analysis, data from three independent qPCR results were calculated by the 2^−ΔΔCt^ method and represented as the means ± S.D. *P* values were determined using Student’s *t* test in each comparison or by one-way analysis of variance (ANOVA) followed by Tukey’s multiple comparison test in more than two groups. Only *P* values lower than 0.05 were considered to be significant.

## Results

### Expression profile of lncRNA in MDA231 cells in response to IMP1 expression

**Fig. 1:**
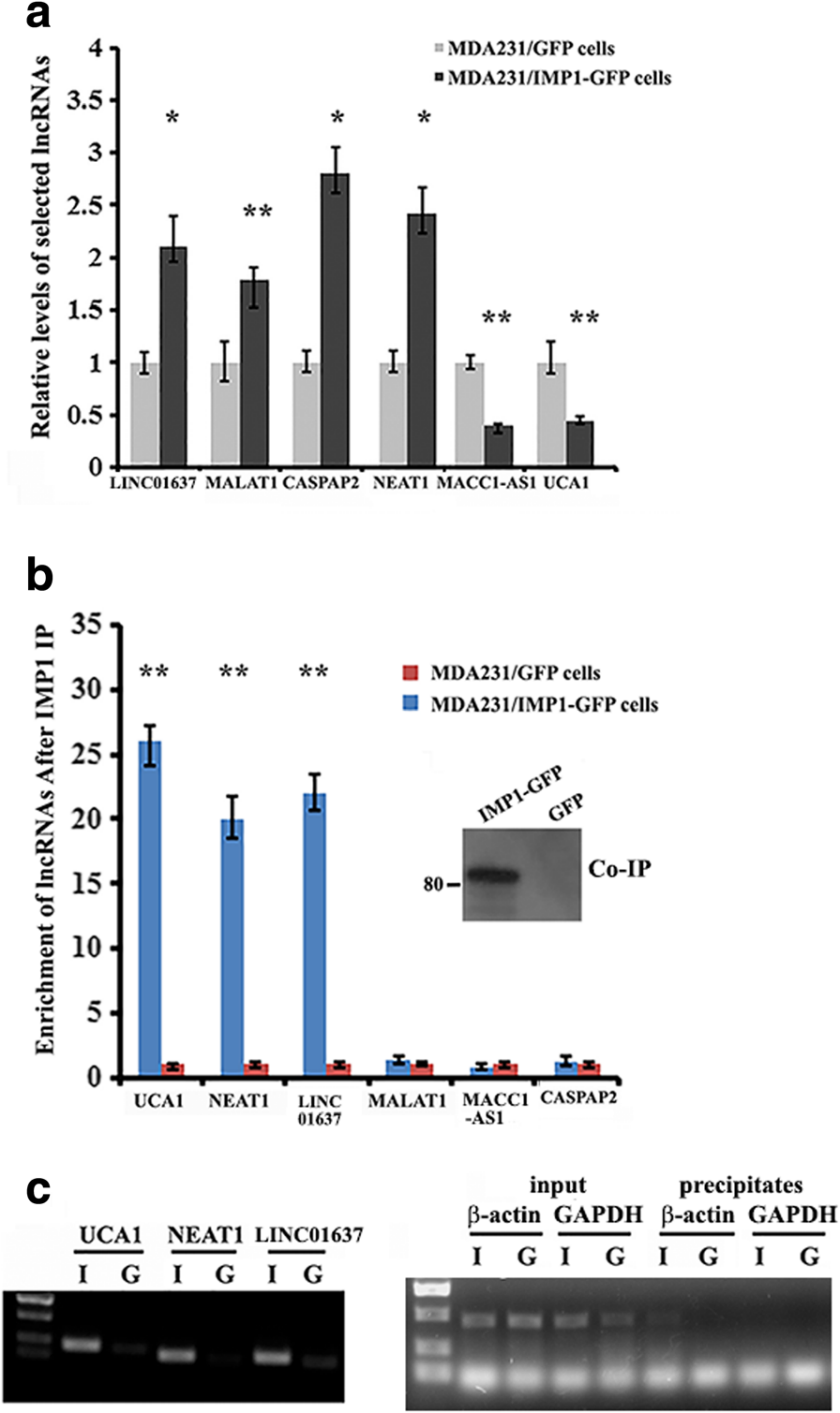
Differential expression of selected microarray-identified long non-coding RNAs (lncRNAs) and their binding to insulin-like growth factor 2 messenger RNA binding protein (IMP1). **a** Total RNA was extracted from MDA231 cells expressing green fluorescent protein (GFP) or Flag-tagged IMP1-GFP. RT-qPCR was used to analyze the levels of six microarray-identified lncRNAs. Relative levels of the lncRNAs were nomalized to glyceraldehyde-3-phosphate dehydrogenase (GAPDH) messenger RNA (mRNA) and statistically analyzed. The data are presented as means ± SD from three independent experiments: *P < 0.05, **P < 0.01 as determined by Student’s *t* test. **b** RNA immunoprecipitation (RIP) was performed to analyze IMP1 interaction with selected lncRNAs. Following IMP1 immunoprecipitation (IP), RNA was extracted and the levels of lncRNAs were measured by RT-qPCR and normalized to GAPDH mRNA levels. Aliquots of the precipitates were used for western blots (inset) to show precipitated IMP1-GFP: **P < 0.01. **c** Selective lncRNAs in the precipitates of individual immunoprecipitates (IPs) were analyzed by RT-PCR followed by agarose gel electrophoresis (left). β-actin mRNA and GAPDH mRNA were used as positive and negative controls for IMP1 RIP (right). I, MDA231/IMP1-GFP cells; G, MDA231/GFP cells

IMP1 has been implicated in many aspects of mRNA regulation [30]. We hypothesized that IMP1 could also be involved in the regulation of lncRNAs in breast cancer cells. To address this, we used lncRNA microarray chips to examine expression profiles of lncRNAs in MDA231/GFP (with lower endogenous IMP1 expression) and MDA231/Flag-IMP1-GFP (IMP1 overexpressing) cells [36]. A total of 1307 lncRNAs with at least a twofold change between the two cell lines were identified, in which 892 genes were upregulated and 415 genes were downregulated in response to IMP1 expression (Additional file 2: Table S3). Of particular interest in the lncRNA involved in tumor progression, we selected four upregulated lncRNAs (long intergenic non-protein coding RNA 1637 (LINC01637) (also named XXbac-B135H6), metastasis-associated lung adenocarcinoma transcript 1 (MALAT1), caspase-8 associated protein-2 (CASPAP2) and nuclear enriched abundant transcript 1 (NEAT1)) and two downregulated lncRNAs (UCA1 and metastasis associated in colon cancer 1-antisense RNA 1 (MACC1)-AS1) to verify their differential expression. qRT-PCR indicated that the expression pattern of the selected lncRNAs was consistent with the microarray results (Fig. 1a). To determine whether the expression changes resulted from the physical interaction between IMP1 and microarray-identified lncRNAs, we performed ribonucleoprotein immunoprecipitation (RIP) assays with antibody against IMP1 and measured the relative levels of the lncRNAs in individual IP samples. NEAT1, UCA1 and LINC01637 lncRNAs were highly enriched in the immunoprecipitates of MDA231/IMP1-GFP cells in contrast to that in MDA231/GFP cells, while the relative levels of the other three lncRNAs in individual IPs were unchanged (Fig. 1b). RT-PCR of selected lncRNAs in the individual precipitates, followed by agarose gel electrophoresis confirmed co-precipitation of IMP1 with UCA1, NEAT1 and LINC01637 lncRNAs. The positive control (β-actin mRNA) and negative control (GAPDH mRNA) for the IMP1 co-IP are also shown (Fig. 1c). These results indicate that IMP1 selectively binds to lncRNAs in breast cancer cells.

### Identification of UCA1 as an IMP1-binding target

**Fig. 2:**
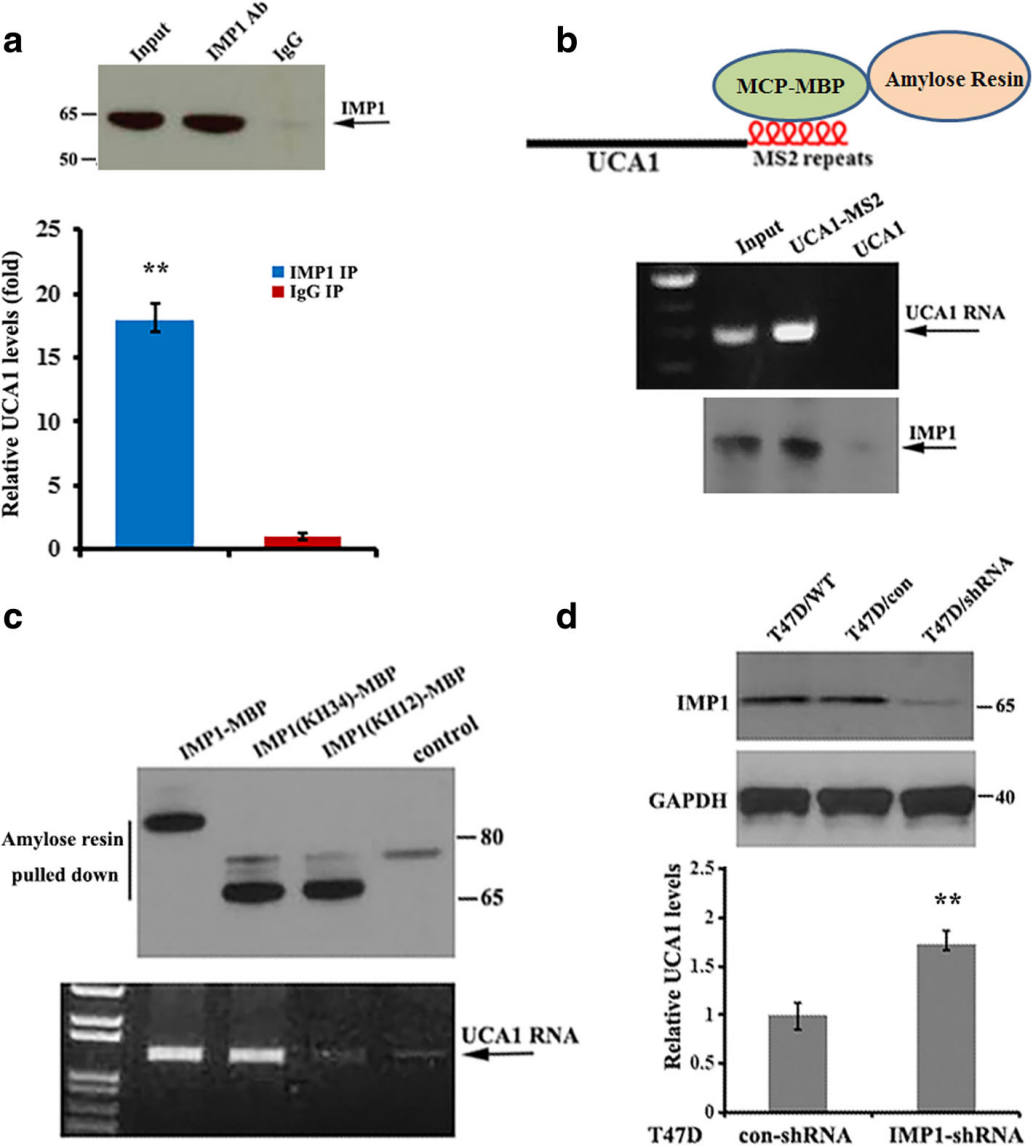
Interaction of insulin-like growth factor 2 messenger RNA binding protein (IMP1) with urethral carcinoma-associated 1 (UCA1) in breast cancer cells. **a** Upper: representitive western blots indicate the precipitation (IP) of IMP1 in T47D cells from co-IP experiments; lower: RT-qPCR shows that UCA1 was preferentially co-precipitated with IMP1 antibody relative to the IgG control. Data are normalized to glyceraldehyde-3-phosphate dehydrogenase (GAPDH) messenger RNA (mRNA) and statistically analyzed: **P < 0.01. **b** Upper: shows the UCA1-MS2 chimeric RNA, which can be bound by MBP-MCP. Lower: UCA1-MS2 was pulled down by MBP-MCP conjugated to amylose resin. Co-precipitated UCA1-MS2 and IMP1 are shown by RT-PCR and western blot analyses. **c** Empty vector (control) and vectors expressing MBP-fused full-length IMP1, IMP1(KH34) or IMP1(KH12) were transiently transfected into 293 T cells. Pulldown assays with amylose resin and western blotting were performed to detect co-precipitation of IMP1 truncates with UCA1. Results show that the full-length IMP1 and IMP1(KH34) co-precipitated with UCA1 in the cell extracts. **d** Knockdown of IMP1 in T47D cells increased stability of UCA1. Upper: immunoblots showing expression of IMP1 in T47D/IMP1-shRNA and control cells. Lower: RT-qPCR indicating levels of UCA1 in IMP1-downregulated cells. Relative levels of UCA1 RNA are normalized to GAPDH mRNA and the data are presented as means ± SD from three independent experiments: **P < 0.01. **P < 0.01 as determined by Student’s *t* test

To study the biological consequence of IMP1-associated lncRNAs, we selected UCA1 for further characterization. Three UCA1 isoforms of 1.4, 2.2 and 2.7 kb in length have been detected in human tumors [39], among which the 1.4-kb isoform is a major isoform in breast cancer cells and has been shown to promote breast tumor progression [15]. In addition to MDA231 cells that overexpressed exogenous IMP1 (Fig. 1b and c), UCA1 was also highly enriched in the IMP1 RIP samples of less invasive T47D cells (Fig. 2a) in which there was higher endogenous IMP1 expression [37]. To further confirm the direct interaction between IMP1 and UCA1 in vivo, we constructed a chimeric UCA1 reporter tagged with six MS2 hairpin repeats (UCA1-MS2, Fig. 2b, upper) and established two stable T47D cell lines expressing UCA1 or UCA1-MS2 RNA (Additional file 3: Figure S1). We then used a recombinant fusion protein (MBP-MCP) that contains a maltose-binding domain (MBP) and a domain that recognizes the MS2 hairpins (MCP) to pull down UCA1 and its associated proteins. RT-PCR (Fig. 2b, middle) and western blots indicated that IMP1 effectively co-precipitated with UCA1-MS2 (Fig. 2b, lower). IMP1 contains multiple RNA-binding domains, in which the KH3 and KH4 domains (termed the KH34 domain) have been previously reported to bind to β-acting mRNA [38, 40]. To identify the domain of IMP1 that was responsible for UCA1 binding we transiently transfected vectors expressing MBP-fused full-length IMP1 or IMP1 truncation mutants into 293 T cells and pulled down MBP-IMP1 fusion proteins with amylose resin from extracts of transfected cells (Fig. 2c, upper). We found that full-length IMP1 and IMP1-KH34, but not the IMP1-KH12, co-precipitated with UCA1 RNA, indicating that the KH34 domain of IMP1 binds to UCA1 (Fig. 2c. lower). We then determined the consequence of IMP1 on UCA1 expression in T47D cells. We observed that the cytoplasmic levels of UCA1 were greatly increased in IMP1 downregulated T47D cells (Fig. 2d).

### Binding of IMP1 destabilizes UCA1

**Fig. 3:**
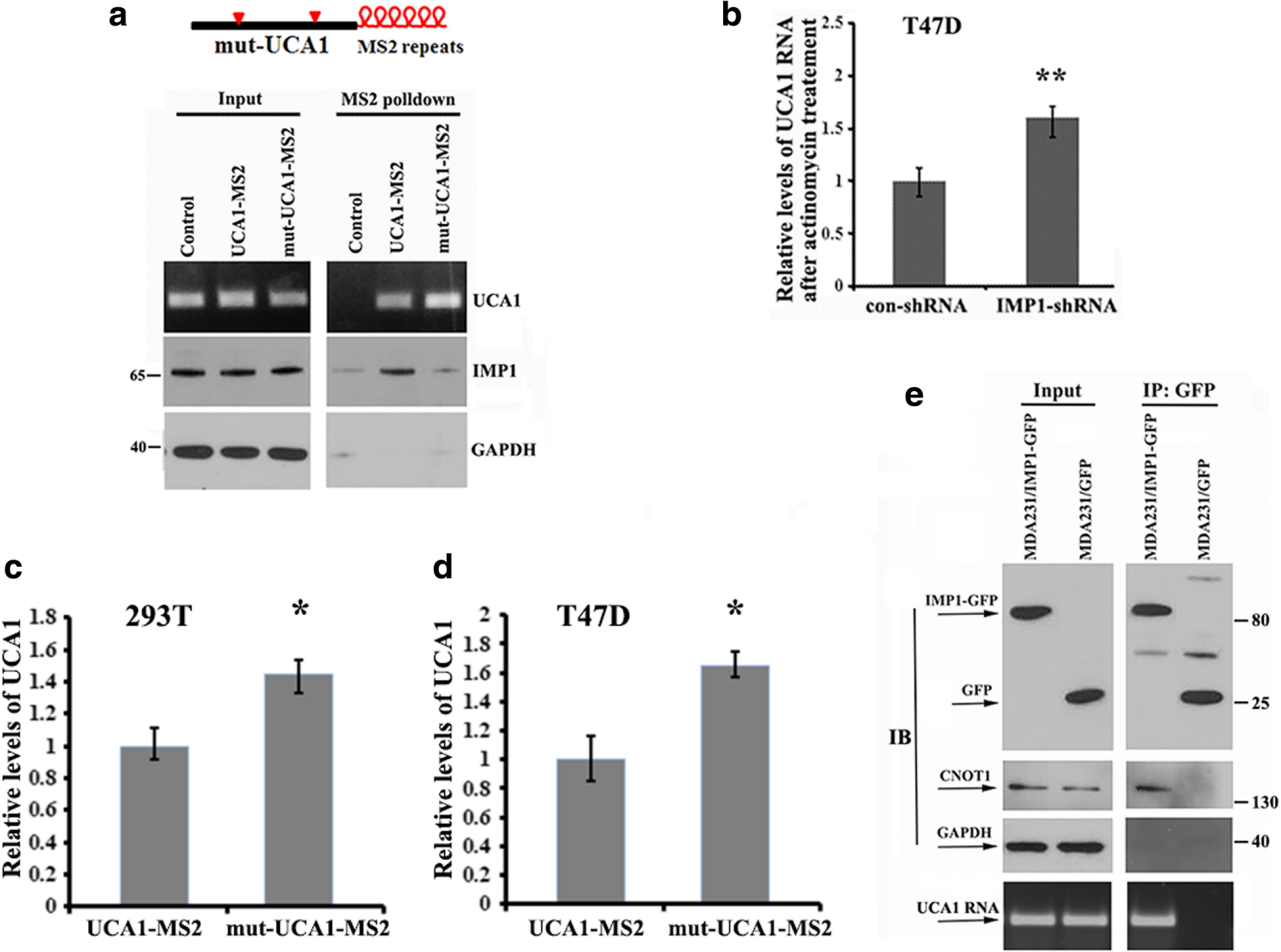
Insulin-like growth factor 2 messenger RNA binding protein (IMP1) binds to urethral carcinoma-associated 1 (UCA1) and decreases UCA1 stability. **a** Upper: shows MS2-UCA1 fusion RNA. UCA1 with mutated IMP1 binding sites are shown by red arrow heads. Lower; UCA1 RNAs were pulled down by MBP-MCP; immunoblots show that interaction of IMP1 with UCA1 was greatly reduced when the IMP1 binding motifs were mutated. Glyceraldehyde-3-phosphate dehydrogenase (GAPDH) was used as a control. **b** Stability of UCA1 in T47D and T47D-IMP1/short hairpin RNA (shRNA) cells were measured after treatment with actinomycin D for 12 h. Relative UCA1 levels were determined by normalizing to GAPDH messenger RNA (mRNA) levels. Data are presented as means ± SD from three independent experiments: **P < 0.01 as determined by Student’s *t* test. **c** and **d** Vectors expressing a green fluorescent protein (GFP) and UCA1 or mutated (mut)-UCA1 were transiently transfected into 293 T and T47D cells for 24 h. Relative levels of UCA1 were analyzed by RT-qPCR and were nomalized to control GFP mRNA levels. The data are presented as mean ± SD from three independent experiments: *P < 0.05. **d** and **e** IMP1 interacts with the components of the RNA decay machinery. Immunoprecipitation was performed on MDA231/IMP1-GFP and MDA231/GFP cells with GFP antibodies. The representative immunoblots (IB) show that IMP1 and UCA1 co-precipitate with CNOT1

Previous studies have shown that IMP1 binds to its target mRNA through the recognition of a conserved ACACCC motif [33, 34]. Interestingly, there are two ACACCC motifs within the UCA1 (Additional file 4: Figure S2A, upper). To determine whether these two motifs were responsible for IMP1 binding, we used PCR-directed mutagenesis to generate a UCA1 mutant (mut-UCA1-MS2), in which both ACACCC motifs within UCA1 were mutated to ACGCTC (Additional file 4: Figure S2A, lower): 293 T cells were then transfected with the constructs expressing wild-type or mutant UCA1 and subjected to pulldown assays using MBP-MCP. IMP1 preferentially co-precipitated with the wild-type UCA1-MS2. In contrast, much lower levels of IMP1 were detected in the precipitates of mut-UCA1-MS2 cells (Fig. 3a). However, when either one of the two motifs was mutated (mut(a)-UCA1-MS2 or mut(b)-UCA1-MS2), IMP1 was still co-precipitated with the single site-mutated UCA1 (Additional file 4: Figure S2B). These results suggest that binding of IMP1 to UCA1 requires at least one of the ACACCC motifs.

Since IMP1 expression resulted in decreased levels of UCA1 in breast cancer cells, we investigated whether IMP1 could regulate UCA1 stability. T47D and T47D/IMP1-shRNA cells were incubated with actinomycin D (5 μg/ml) for 12 h to block de novo transcription, and the endogenous levels of UCA1 were measured by RT-qPCR. Results showed that, compared to control cells, reducing IMP1 expression increased levels of UCA1 RNA (Fig. 3b). To determine whether UCA1 decay requires the interaction of IMP1 with UCA1, we separately transfected vectors expressing UCA1-MS2 or mut-UCA1-MS2 into 293 T cells and analyzed their expression. Since the vector also expresses a GFP marker, the GFP mRNA was used as an internal control for RT-qPCR. In comparison to wild-type UCA1-MS2, the levels of mut-UCA1 RNA were significantly increased (Fig. 3c). Similar results were also observed in T47D cells transfected with UCA1-MS2 or mut-UCA1-MS2-expressing vectors (Fig. 3d). These experiments indicate that interaction between IMP1 and UCA1 reduced the stability of UCA1. Thus, we hypothesized that IMP1 could be associated with the RNA decay machinery in mediating UCA1 degradation. To address this hypothesis, we investigated the potential interaction of IMP1 and UCA1 with components of the CCR4-NOT complex. Co-IP experiments using antibody against GFP in MDA231/GFP and MDA231/IMP1-GFP cells indicated that both UCA1 and CNOT1, a scaffold protein of the CCR4-NOT complex responsible for poly(A) tail shortening and subsequent RNA decay [41], were enriched in IMP1-GFP precipitates (Fig. 3d). A reciprocal pulldown experiment has also shown that UCA1-MS2 was associated with CNOT1 in MDA231/IMP1-GFP cells (Additional file 5: Figure S3A), suggesting that IMP1 may recruit UCA1 into CCR4-NOT complexes. Interestingly, a recent study has also reported that in liver cancer cells IMP1 could be associated with the CCR4-NOT RNA decay machinery in enhancing the degradation of its associated lncRNA HULC [29].

### Identification of miRNAs that bind to UCA1 in breast cancer cells

**Fig. 4:**
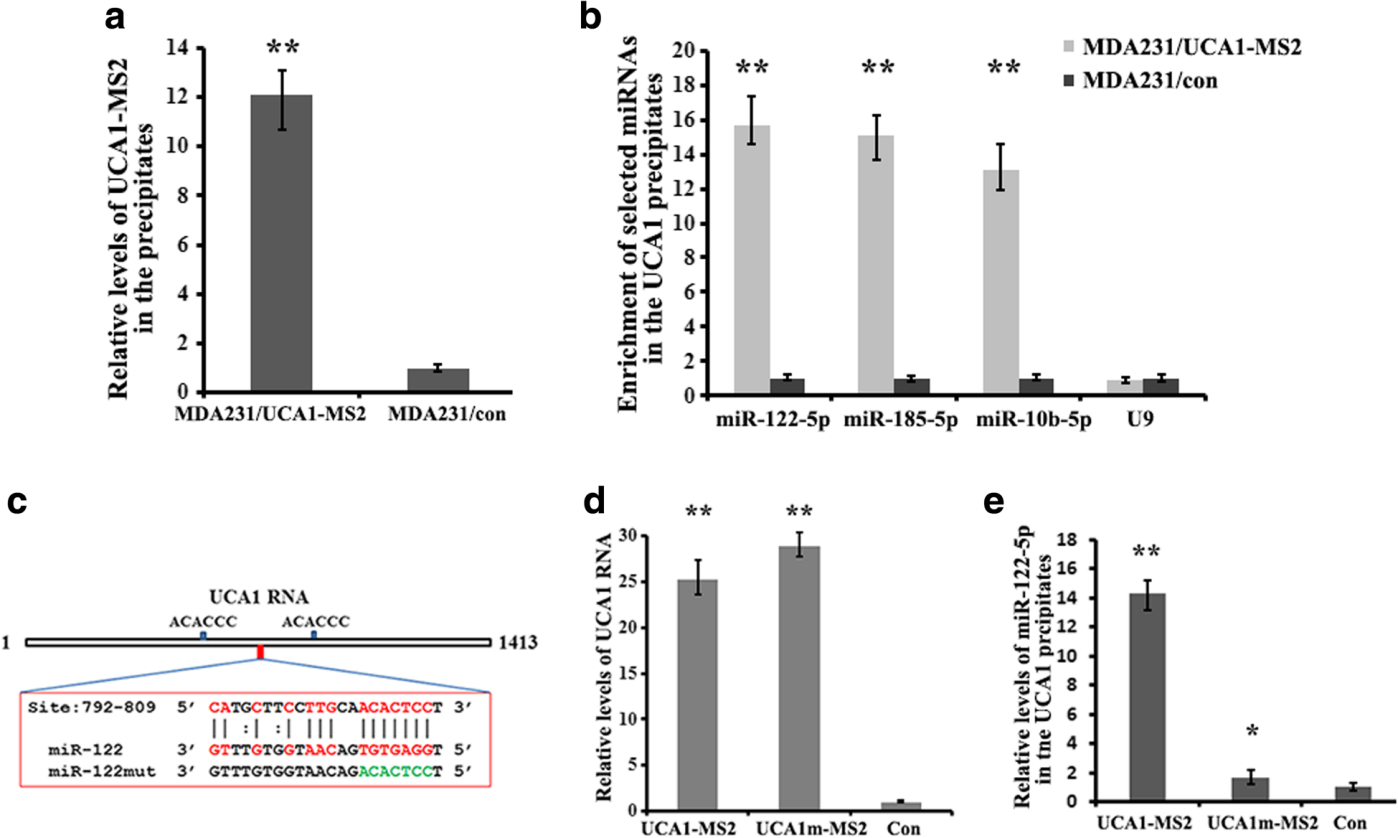
Identification of urethral carcinoma-associated 1 (UCA1)-associated micro RNAs (miRNAs). **a** MS2 pulldown was performed in MDA231/UCA1-MS2 cells. Enrichment of UCA1-MS2 in the precipitates was measured by RT-qPCR: **P < 0.01. **b** After MS2 pulldown, the levels of selected miRNAs associated with UCA1 were measured by RT-qPCR. The data are presented as means ± SD from three independent experiments: **P < 0.01 as determined by Student’s *t* test. **c** Shows the putative binding sites for insulin-like growth factor 2 messenger RNA binding protein (IMP1) and miR-122-5p. The mutated miR-122-5p binding site on UCA1 is shown as green. **d** At 24 h after transfection with either UCA1-MS2 or UCA1m-MS2 construct, 293 T cells were lysed and the lysates were analyzed by pulldown assays using MBP-MCP-conjugated amylose resin. Both MS2-tagged UCA1 and UCA1m RNAs were precipitated. **e** Interaction of miR-122-5p with UCA1-MS2 was examined in the pulldown material. Relative levels of miR-122-5p in the precipitates are represented as mean ± SD from three independent experiments: *P < 0.05, **P < 0.01

To explore the biological significance of IMP1-regulated UCA1 expression, we precipitated MS2 hairpin-tagged lncRNA UCA1 in breast cancer cells to identify UCA1-associated miRNAs. After extraction of RNA from the precipitates, an aliquot of the RNA was used for RT-qPCR to verify precipitated UCA1-MS2 (Fig. 4a), and the other was sent to Guangzhou RiboBio Co. (China) for microRNA sequencing for miRNAs associated with UCA1 (the data have been deposited in the GEO [GEO:GSE92319]. The sequencing data indicated that miRNAs, such as miR-122-5p, miR-185-5p and miR-10b-5p, which are known to be involved in inhibition of cell invasion and proliferation [42–44], were preferentially enriched in the UCA1-MS2 pulldown material compared to control cells (Additional file 6: Table S2). Binding of UCA1 to the three miRNAs was confirmed by RT-qPCR in the UCA1 pulldown materials (Fig. 4b). Analysis using the online bioinformatics tool (microRNA.org-target program) identified that UCA1 contains putative binding sequences complementary to the seed regions of miR-122-5p (Fig. 4c) as well as miR-185-5p (Additional file 5: Figure S3B). We hereafter selected miR-122-5p for further investigation. We mutated the putative binding site for miR-122-5p within the UCA1 sequence (denoted as UCA1m, Fig. 4c) and then transfected WT-UCA1-MS2 or UCA1m-MS2 vector into 293 T cells, respectively. Pulldown experiments followed by RT-qPCR analyses showed that although similar levels of WT-UCA1 and UCA1m RNAs were precipitated (Fig. 4d), association between miR-122-5p and UCA1m-MS2 was significantly reduced (Fig. 4e). miR-185-5p was also enriched in the precipitates of WT-UCA1-transfected 293 T cells (Additional file 5: Figure S3C). These results indicate the physical association between UCA1 and miR-122-5p and miR-185-5p in vivo.

### UCA1 serves as a sponge for miR-122-5p

**Fig. 5:**
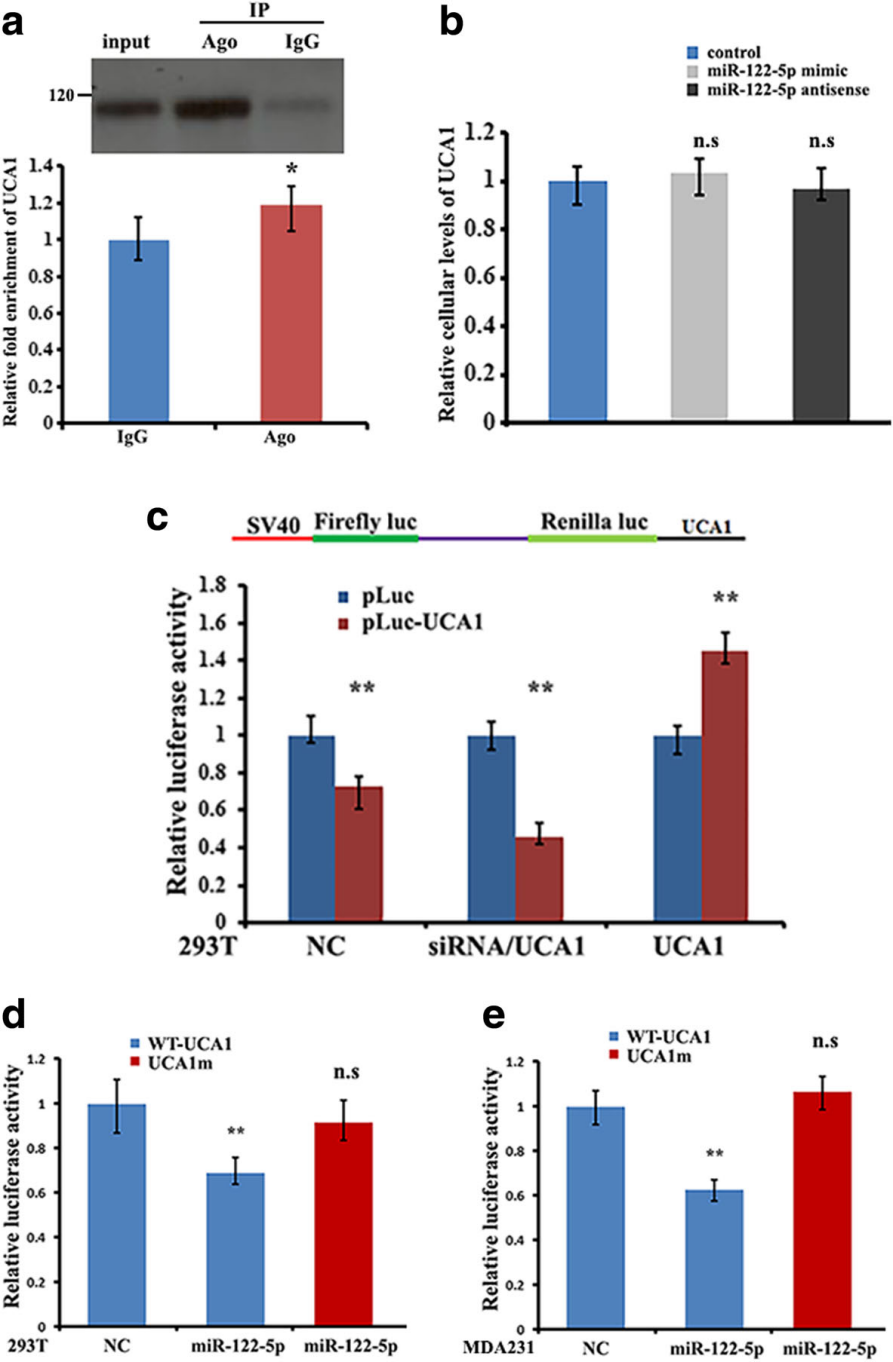
Urethral carcinoma-associated 1 (UCA1) functions as a miR-122-5p sponge. **a** Upper: antibody pulldown of Ago2 protein. Lower; UCA1 levels in the immunoprecipitates of Ago2 were measured by RT-qPCR: *P < 0.05. **b** RT-qPCR analysis shows the relative levels of UCA1 in MDA231 cells after ectopic expression of miR-122-5p and antisense-oligonucleotides to miR-122-5p: n.s, not significant. **c** Upper: the entire UCA1 sequence was cloned into the downstream region of the renilla luciferase gene of the PsiCHECK 2 dual luciferase reporter construct (denoted as pluc-UCA1). Lower: 293 cells were transfected with pluc-UCA1 (NC), or pluc-UCA1 in combination with the UCA1-siRNA or UCA1 vector. Luciferase activity was determined by luciferase reporter assays. Activity of Renilla luciferase was normalized to the activities of firefly luciferase: **P < 0.01 as determined by Student’s *t* test. **d** and **e** The 293 T and MDA231 cells were transfected with pluc-UCA1 reporter (NC) or co-transfected with mature miR-122-5p mimics in combination with pluc-UCA1 reporter or the reporter harboring mutated miR-122-5p binding site (pluc-UCA1m). Relative luciferase activity is presented as means ± SD from three independent experiments: **P < 0.01; n.s, not significant; WT, wild-type

The main function of miRNA is to downregulate gene expression by binding to complementary sites of their RNA targets within a RNA-induced silencing complex (RISC) [45, 46]. LncRNAs frequently act as sponges to sequester miRNAs and thus affect the expression of downstream targets of the miRNAs [12, 13, 47]. To test whether binding of miRNAs to UCA1 would result in UCA1 associating with RISC, a RIP experiment was performed in the extracts of MDA231 cells using antibody against Ago2, a core component of RISC, and was followed by RT-qPCR analysis. The result indicated that UCA1 was only slightly enriched in Ago2-containing precipitates compared to the control IgG precipitates (Fig. 5a). In addition, overexpression of miR-122-5p mimic or antisense-oligonucleotides to miR-122-5p did not change the cellular levels of UCA1 (Fig. 5b), suggesting that association of miR-122-5p does not affect UCA1 stability. We then constructed two luciferase reporters, in which the sequence of UCA1 (pluc-UCA1) or UCA1m (pluc-UCA1m) was inserted downstream of the Renilla luciferase gene (Fig. 5c, upper). Inclusion of the UCA1 sequence in the 3’UTR reduced luciferase activity. Co-transfection with UCA1-siRNA further decreased luciferase activity, while overexpressing UCA1 increased luciferase activity (Fig. 5c lower). Subsequent co-transfection of the miR-122-5p mimic with the pluc-UCA1 reporter into 293 T and MDA231 cells repressed luciferase activity. In contrast, co-transfection of the miR-122-5p mimic with pluc-UCA1m reporter indicated that the mutated binding site for miR-122-5p abolished the repressive effect of miR-122-5p (Fig. 5d and e). Taken together, these results suggest that UCA1 could function as a sponge for miR-122-5p.

### Competitive binding of IMP1 to UCA1 releases miR-122-5p and increases the efficiency of miR-122-5p to regulate its target mRNAs

**Fig. 6:**
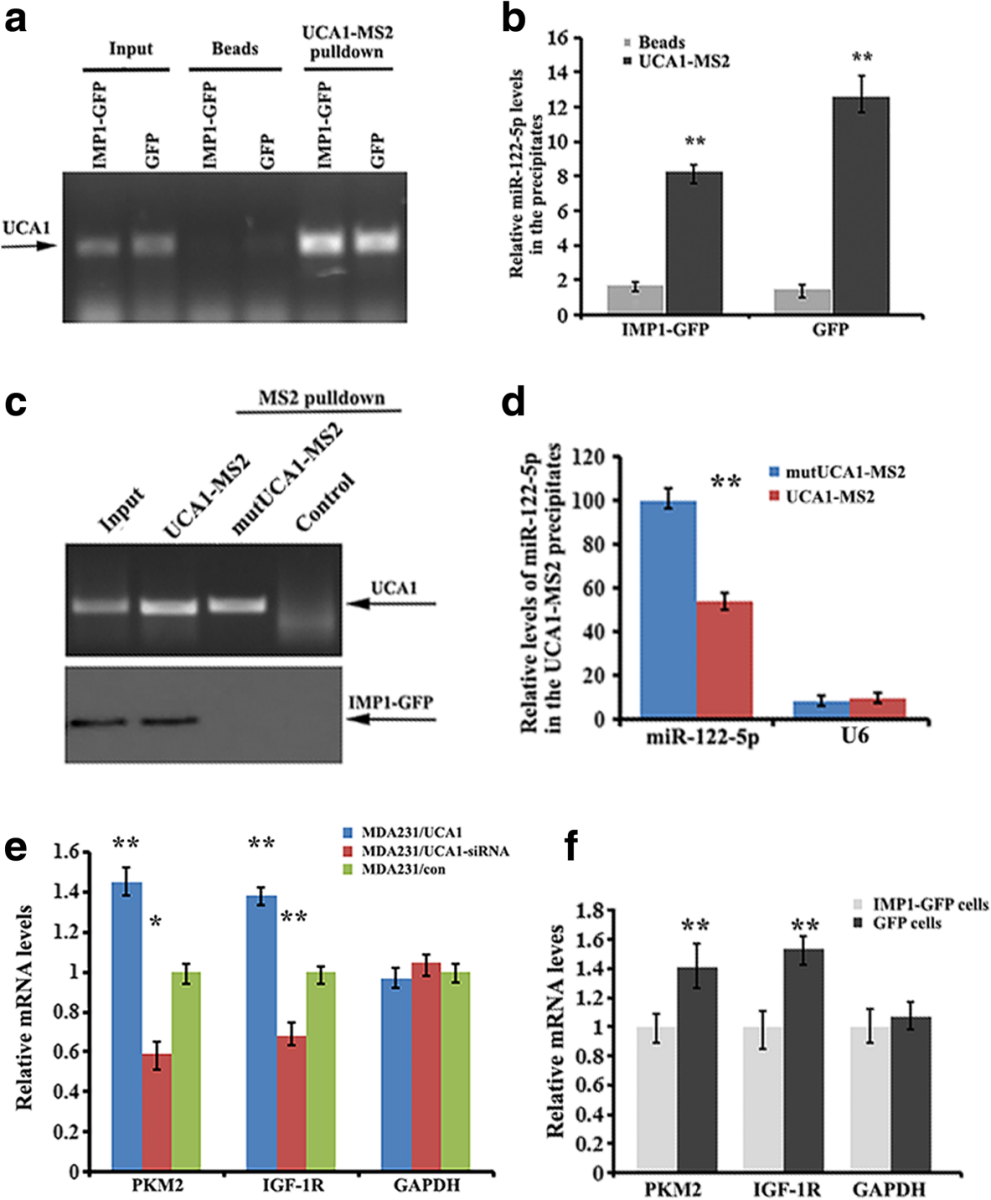
Competitive binding of insulin-like growth factor 2 messenger RNA binding protein (IMP1) to urethral carcinoma-associated 1 (UCA1) decreases the binding of miR-122-5p to UCA1. **a** Lentivirus vector expressing UCA1-MS2 was infected into MDA231/IMP1-green fluorescent protein (GFP) and MDA231/GFP cells. MS2 pulldown followed by RT-PCR analysis indicates the precipitated UCA1-MS2. **b** Enrichment of miR-122-5p in the precipitates of UCA1-MS2 was measured by RT-qPCR. IMP1 expression decreased binding of miR-122-5p to UCA1: **P < 0.01 as determined by Student’s *t* test. **c** MS2 pulldown analysis showed that UCA1 with muatated IMP1 binding motifs does not bind to IMP1. **d** RT-qPCR indicates that the UCA1-IMP1 interaction decreased the association of UCA1 with miR-122-5p. **e** Levels of pyruvate kinase muscle isozyme M2 (PKM2), insulin-like growth factor 1 receptor (IGF-1R) and glyceraldehyde-3-phosphate dehydrogenase (GAPDH) messenger RNAs (mRNAs) were measured in MDA231/UCA1, MDA231/UCA1-small interfering RNA (siRNA) and MDA231 control cells. Relative levels of mRNAs are the means ± SD from three independent experiments: **P < 0.01, *P < 0.05 as determined by one-way analysis of variance followed by Tukey’s multiple comparison tests. **f** RT-qPCR was used to monitor the effect of IMP1 overexpression on the expression levels of PKM2 and IGF-1R mRNAs. GAPDH mRNA was used as an internal control; means ± SD from three independent experiments: **P < 0.01 as determined by Student’s *t* test

The miR-122-5p binding site on UCA1 is located in the middle of the two IMP1 binding sites (Fig. 4c). Based on the experimental results that UCA1 could interact with both IMP1 and miR-122-5p, we postulated that IMP1 expression could change the extent of the UCA1/miR-122-5p interaction. To address this, we compared the binding efficiency of UCA1 to miR-122-5p in the presence of IMP1. The IMP1 expression did not affect cellular levels of miR-122-5p in MDA231/GFP and MDA231/IMP1-GFP cell lines (Additional file 7: Figure S4A). However, weak enrichment of miR-122-5p was shown in the UCA1-pulldown material of MDA231/IMP1-GFP cells in comparison to increased association between miR-122-5p and UCA1 in MDA231/GFP cells (Fig. 6a and b). Moreover, after pulldown assays, the levels of miR-122-5p were obviously higher in the cell supernatant of IMP1-expressing cells in contrast to the cells without exogenous IMP1 expression (Additional file 7: Figure S4B). To further confirm the effect of competitive binding of IMP1 to UCA1 for miR-122-5p release, 293 T cells expressing the wild-type UCA1-MS2 or IMP1 binding site-mutated UCA1 (mut-UCA1-MS2) were subjected to MS2 pulldown. In this case, since IMP1 was not able to interact with mut-UCA1, miR-122-5p displayed stronger association with mut-UCA1-MS2 than with wild-type UCA1 (Fig. 6c and d). These results suggest that binding of IMP1 to UCA1 reduces the sponge effect of UCA1 on miR-122-5p.

Given that UCA1 acts as a sponge for miR-122-5p and IMP1 decreases the UCA1 sponge effect, it is possible that alteration of either UCA1 or IMP1 expression could eventually affect the fate of those mRNAs that are regulated by miR-122-5p. As shown in Fig. 6e, increased expression of UCA1 enhanced, while downregulation of UCA1 decreased expression of pyruvate kinase muscle isozyme M2 (PKM2) and IGF-1R mRNAs, which are endogenous targets of miR-122-5p. In contrast, overexpression of IMP1 in MDA-MB-231 cells reduced the expression of PKM2 and IGF-1R mRNAs, presumably from a decreased sponge effect of UCA1 to miR-122-5p (Fig. 6f). Conversely, silencing IMP1 in T47D cells increased the levels of PKM2 and IGF-1R mRNAs (Additional file 7: Figure S4C).

### IMP1 represses UCA1-mediated breast cancer cell invasion

**Fig. 7:**
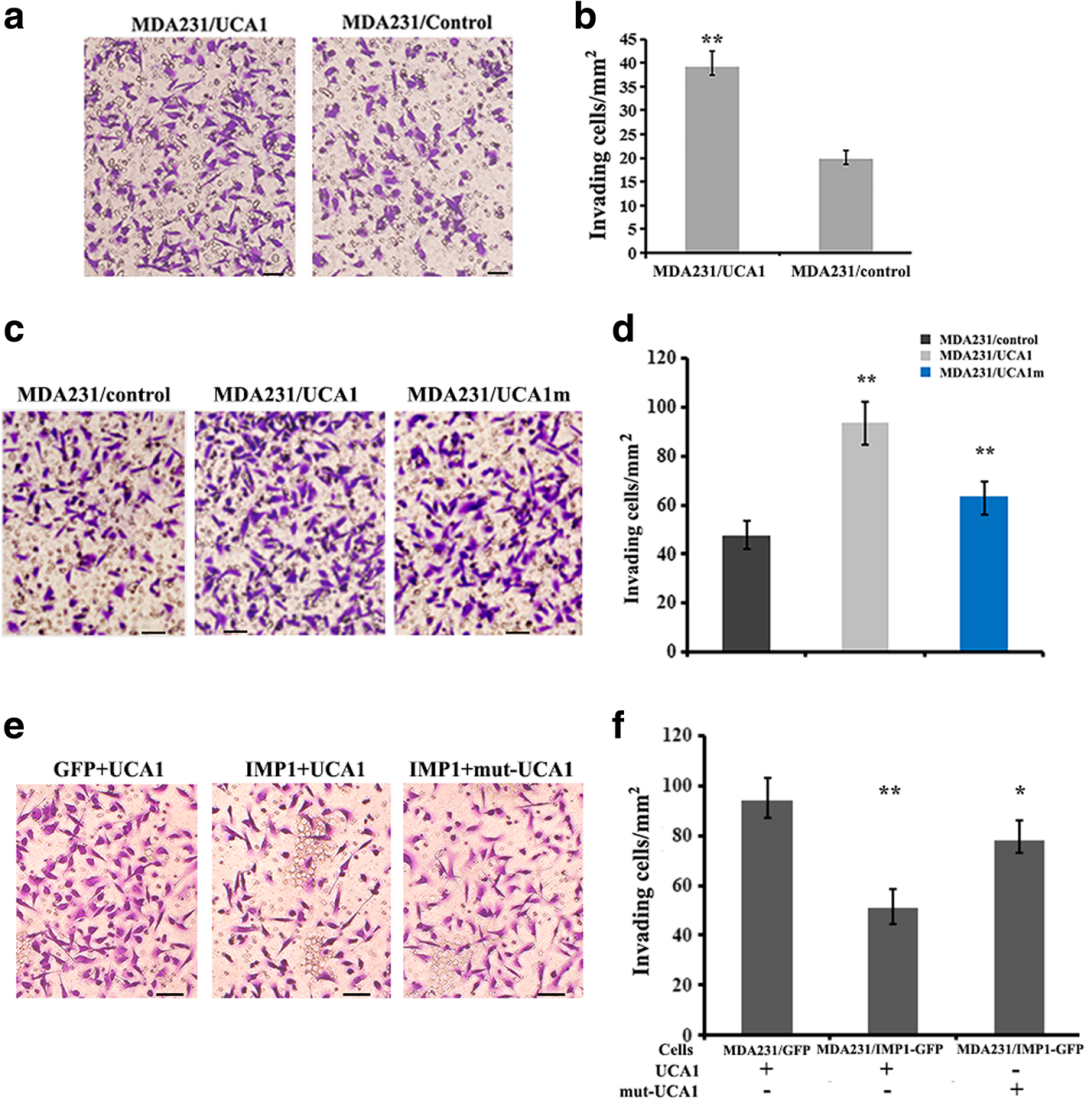
Insulin-like growth factor 2 messenger RNA binding protein (IMP1) inhibits urethral carcinoma-associated 1 (UCA1)-mediated cancer cell invasion. MDA231 cells suspended in 1% fetal bovine serum medium were plated into the upper chamber of 8-mm-pore Matrigel-coated transwell filters. The lower chamber contained medium with 10% fetal bovine serum. Cells that had invaded to the underside of the filter were stained and counted. **a** Representative images of transwell assays in control (MDA231/con) and UCA1 overexpressing (MDA231/UCA1) cells. **b** Effect of UCA1 on cell invasive abilities. Values represent the means ± SD from three independent experiments: **P < 0.01 as determined by Student’s *t* test. **c** Wild-type (WT)-UCA1 and UCA1m (UCA1 with a mutated miR-122-5p binding site) were expressed in MDA231 cells. Representative images of transwell assays are shown. **d** Invasive ability of the cells was analyzed. Relative numbers indicate the mean ± SD from three independent experiments: **P < 0.01 as determined by one-way analysis of variance (ANOVA) followed by Tukey’s multiple comparison tests. **e** UCA1 and mut-UCA1 (UCA1 with mutations in the two IMP1-binding motifs) were expressed in MDA231/green fluorescent protein (GFP) and MDA231/IMP1-GFP cells. Representative images of transwell assays are shown. **f** Invasive ability of the cells was measured by transwell experiments. Relative numbers indicate the means ± SD from three independent experiments: **P < 0.01, *P < 0.05 as determined by one-way ANOVA followed by Tukey’s multiple comparison tests

We next examined the biological function of IMP1 in the regulation of UCA1-mediated breast cancer cell invasion. Ectopic expression of UCA1 significantly enhanced the invasive ability of MDA-MB-231 cell (Fig. 7a and b). Similar results were observed in MCF7 cells, in which overexpression of UCA1 increased, while downregulation of UCA1 decreased cell invasiveness (Additional file 8: Figure S5A). However, mutation of the binding site for miR-122-5p (UCA1m) greatly attenuated the effect of UCA1 on cell invasion, most likely from the loss of UCA1/miR-122-5p interaction (Fig. 7c and d). Blocking of the UCA1/miR-122-5p interaction also reduced cell proliferation (Additional file 8: Figure S5B). Furthermore, when IMP1 was expressed in UCA1-overexpressing MDA231 cells, the invasive effect of UCA1 was largely reduced. In comparison, the invasive ability of UCA1 was still higher when the IMP1 binding motifs of UCA1 were mutated (Fig. 7e and f). These results indicate that IMP1 suppresses UCA1-mediated cell invasion.

Thus, we propose a model in which IMP1 regulates the sponge effect of UCA1 for miR-122-5p (Additional file 9: Figure S6). In breast cancer cells, lower IMP1 expression allows UCA1 to stably associate with miR-122-5p (S6A). Increasing IMP1 expression results in IMP1 binding to UCA1, thereby reducing UCA1/miR-122-5p interaction by competing with UCA1 for miR-122-5p binding (S6B). By association directly or indirectly with CNOT1, IMP1 recruits the CCR4-NOT deadenylase complex onto UCA1 and causes UCA1 decay (S6C). This leads to increases in miR-122-5p availability, permitting miR-122-5p to bind to target mRNAs and assert its post-transcriptional function (S6D).

## Discussion

We have identified a lncRNA UCA1 that interacts with IMP1 and have characterized the biological consequences of this interaction. We show that IMP1 binds to the ACACCC motifs of UCA1 via the KH34 domain of the protein. Binding of IMP1 not only destabilizes UCA1 through the recruitment of the CCR4-NOT deadenylase complex, but also decreases the association between UCA1 and miRNAs, including miR-122-5p, therefore shifting the miRNA from UCA1 to its target mRNAs and leading to increased availability of miR-122-5p to suppress its target gene expression.

LncRNAs can normally function as competitive endogenous RNA (ceRNA) to modulate post-transcriptional regulation by integrating with miRNAs and regulating expression of miRNA target genes [13]. These lncRNAs usually contain miRNA responsive elements (MREs) and function as miRNA sponges to regulate endogenous miRNAs, thus reducing miRNA-induced repression of their target mRNAs [20, 48, 49]. UCA1 was originally reported to be over-expressed in bladder cancer and was suggested to be a biomarker for the diagnosis of bladder tumors [39]. Since then, high expression of UCA1 has been observed in a number of human cancers, including colorectal cancer and breast cancer, suggesting a common oncogenic role of UCA1 in tumorigenesis [15, 50]. Recently, a number of studies have reported the interaction of UCA1 with miRNAs. For example, UCA1 could function as an endogenous sponge for miR-216b to regulate the expression of fibroblast growth factor receptor 1 (FGFR1) and the extracellular related kinase (ERK) signaling pathway in hepatocellular carcinoma [16], for miR-204-5p to upregulate CAMP responsive element binding protein 1 (CREB1) gene expression in colorectal cancer [18] and for miR-122 to promote glioma cell proliferation and invasion [51]. In the present study, we identified association between UCA1 and miR-122-5p in breast cancer cells. Mutation of the MRE on UCA1 abolished the binding ability of miR-122-5p to UCA1 and subsequently affected the expression of miR-122-5p target genes (e.g. IGF-1R, PKM2). However, IMP1 expression reduced the binding of miR-122-5p to UCA1, indicating the regulatory role of IMP1 on UCA1/miR-122-5p interaction. Moreover, in the absence of IMP1, association between miR-122-5p and UCA1 barely affected the UCA1 stability, supporting the conclusion that UCA1 sponges miR-122-5p.

IMP1 is a multifunctional RNA-binding protein that contains four KH domains for target RNA recognition [40]. The KH34 domains of the protein recognize the ACACCC motif within the 3’ UTR of β-actin mRNA [33, 34]. In the study, we also demonstrate that the KH34 domains of IMP1 and the ACACCC motifs within UCA1 are required for IMP1/UCA1 interaction. Either lack of the IMP1/KH34 domains or mutations in the ACACCC motifs of UCA1 dramatically decreases the ability of IMP1 to bind UCA1. These results may suggest a common molecular mechanism for IMP1 to bind to target mRNA or lncRNAs. Our results indicate that in addition to facilitating UCA1 decay by recruitment of the CCR4-NOT1 complex, the IMP1/UCA1 interaction could also compete for miR-122-5p binding to UCA1. Interestingly, the putative miR-122-5p binding site within UCA1 is located in the middle of the two ACACCC motifs, with the upper-stream motif being about 130 nucleotides and the downstream motif being nearly 280 nucleotides away from the miR-122-5p binding site. We postulate that folding of UCA1 generates the structure preferentially for IMP1 binding, which results in IMP1 in proximity to the miR-122-5p binding site, leading to functional blocking of the miR-122-5p association.

miR-122-5p is a tumor suppressor participating in the regulation of several mRNAs [52, 53]. As key targets of miR-122-5p, IGF-1R and PKM2 have been reported to promote tumor growth and metastasis, indicating the functional similarity of UCA1. Our data reveal that UCA1 upregulates the IGF-1R and PKM2 by competitively sponging miR-122-5p, and thus promotes the invasive potential of breast cancer cells. However, this phenotype could be reversed in the presence of IMP1, which not only destabilizes UCA1, but also acts as an endogenous competitor to release the sponge effect of UCA1 on the activity of miR-122-5p. Since IMP1 has been shown to be involved in many cellular processes through the regulation of its mRNA targets [35, 36], this study reveals an additional role of IMP1 to regulate lncRNA and hence establishes the function of IMP1 as a gene regulator in breast cancer.

## Conclusions

Our present study represents a paradigm that the RNA-binding protein IMP1 might serve as a regulator to mediate the decay and the sponge effect of a lncRNA. Binding of IMP1 to UCA1 not only decreases the stability of UCA1, but also modulates the binding ability of miR-122-5p to UCA1. Both of these result in the liberation of miR-122-5p activity and the reduction of cell invasiveness. Alterations in IMP1 or UCA1 expression could shift this competitive balance. Our data indicate a novel model to depict an IMP1/UCA1/miR-122-5p interaction during the progress of breast cancer cell invasion. This mechanism will lead to a better understanding of factors influencing breast cancer cell invasion. Further characterization of the IMP1-lncRNA-miRNA network will provide new insights into the regulation of lncRNA-mediated gene expression.

## Additional files

Additional file 1: **Table S1.** Primers used for qPCR, RT-PCR and siRNA interference. (DOC 53 kb)

Additional file 2: miRNAs associated with UCA1. (XLSX 375 kb)

Additional file 3: **Figure S1.** UCA1 expression in MDA-MB-231 stable cell lines. (A) MDA231 cell lines stably expressing UCA1 or UCA1-MS2 were established. RT-qPCR analysis was performed to verify UCA1 expression. UCA1 levels were normalized to GAPDH mRNA from three independent experiments: **P < 0.01. (B) UCA1 knockdown MDA231 stable cell line was established by a lentivirus expressing UCA1-shRNA. RT-qPCR assays indicated that expression of UCA1 was knocked down by about 60%. Assays were normalized to GAPDH mRNA from three independent experiment:. **P < 0.01. (TIFF 336 kb)

Additional file 4: **Figure S2.** UCA1 sequence and mutagenesis. The putative motifs for IMP1 binding in UCA1 are indicated in red. Mutations of the putative binding sites are shown below the UCA1 sequences. (TIFF 2587 kb)

Additional file 5: **Figure S3.** UCA1 is associated with IMP1 and CNOT1 and with miR-185-5p. (A) Vectors expressing UCA1 or UCA1-MS2 were transiently transfected into MDA231/IMP1-GFP cells. Pulldown assays were performed to analyze the association of IMP1 and CNOT1 with UCA1-MS2. Representative images indicate that both IMP1 and CNOT1 co-precipitated with UCA1. Control: cells transfected with MS2-untagged UCA1. (B) Putative binding site of UCA1 for miR-185-5p. (C) Interaction of miR-185-5p with UCA1-MS2 was examined in the pulldown material. Relative levels of miR-185-5p in the precipitates were statistically analyzed as means ± SD from three independent experiments: **P < 0.01 as determined by Student’s *t* test. (TIFF 1227 kb)

Additional file 6: **Table S2.** miRNAs associated with UCA1. (DOCX 13 kb)

Additional file 7: **Figure S4.** IMP1 knockdown increases the expression of miR-122-5p target mRNAs. (A) Cellular levels of miR-122-5p are not affected by IMP1-GFP expression. (B) After MS2 pulldown experiments, levels of miR-122-5p in the supernatants were analyzed by qPT-PCR. Levels of miR-122-5p were normalized to GAPDH mRNA from three independent experiments: **P < 0.01 as determined by Student’s *t* test. (C) RT-qPCR was applied to measure the levels of PKM2 and IGF-1R mRNAs in IMP1 knockdown T47D cells. Levels of the mRNAs were normalized to GAPDH mRNA from three independent experiments: *P < 0.05 as determined by Student’s *t* test. (TIFF 884 kb)

Additional file 8: **Figure S5.** Effect of UCA1 on the invasive abilities of MCF7 cells. Histograms show the effect of UCA1 on the invasive abilities of MCF7 cells. Values represent the means ± SD from three independent experiments; **P < 0.01, *P < 0.05 as determined by one-way ANOVA followed by Tukey’s multiple comparison tests. (TIFF 992 kb)

Additional file 9: **Figure S6.** A proposed model of IMP1 to regulate the sponge effect of UCA1 for miR-122-5p. (A) UCA1 sponges miR-122-5p, reducing miR-122-5p interaction with target mRNA. (B) Increasing IMP1 expression allows IMP1 to bind to UCA1 and to release miR-122-5p from UCA1. This increases the availability of miR-122-5p to interact with target mRNA. (C) Binding to target mRNA allows miR-122-5p to assert its posttranscriptional function. (D) IMP1 binds to UCA1 and recruits it to the CCR4-NOT1 complex, initiating UCA1 decay process. (TIFF 1116 kb)
