# Supplementary material for: IMP1 regulates UCA1-mediated cell invasion through facilitating UCA1 decay and decreasing the sponge effect of UCA1 for miR-122-5p
Source: Breast Cancer Res. 2018 Apr 18;20:32. doi: 10.1186/s13058-018-0959-1 (PMC5907460; doi:10.1186/s13058-018-0959-1)
Supplement: Supplementary file 1 — Table S1. Primers used for qPCR, RT-PCR and siRNA interference. (DOC 53 kb) [file 13058_2018_959_MOESM1_ESM.doc]

**Table S1 Primers used in this study**

| Primers for PCR | Sequences |
| --- | --- |
| GAPDH Forward | GAGTCAACGGATTTGGTCGT |
| GAPDH Reverse | TGGGATTTCCATTGATGACA |
| UCA1 Forward | ATGGTGTCCTCAAGCCTACT |
| UCA1 Reverse | ATGTCCCAAGCCCTCTAAC |
| UCA1 Forward (RIP) | AGTGGCTGAAGACTGATGCTG |
| UCA1 Reverse (RIP) | TGGTCCATTGAGGCTGTAGAG |
| PKM2 Forward | CTGTGGACTTGCCTGCTGT |
| PKM2 Reverse | TGCCTTGCGGATGAATGACG |
| UCA1-NotI (Cloning) | GCGCGGCCGCTGACATTCTTCTGGACAATG |
| UCA1-BamHI (Cloning) | GCGGATCCTTTATCAGGCATATTAGCTTTAATG |
| MACC1-AS1 Forward | GAACCCTGCACTTGAACAACAC |
| MACC1-AS1 Reverse | CTATTCACAACCTGTTCCTCAT |
| MALAT1 Forward | AGTACAGCACAGTGCAGCTTTG |
| MALAT1 Reverse | CACCAATCCCAACCGTAACAG |
| Neat1 Forward | TGGTAGACAGAATCCATGTACCT |
| Neat1 Reverse | GCCATTGGTATTACTAATCCAATT |
| CASP8AP2 Forward | CTGTTGCTCCAGGCTTTCAGT |
| CASP8AP2 Reverse | TTCATCAGGCATCATACTCTG |
| LINC01637 Forward | ATGTACCGCGTGACGTGAGAGC |
| LINC01637 Reverse | TTTTCAATTATATTTTATTACATTTT |
| IGF1R Forward | TGCTGTATGCCTCTGTGAACC |
| IGF1R Reverse | AGACCATCCCAAACGACCC |
| GFP Forward | GCAAGCTGACCCTGAAGTTCAT |
| GFP Reverse | CCTTGAAGAAGATGGTGCGCTC |
| MS2 Forward | GACACTTCACAATCAAGGGGTAC |
| MS2 Reverse | GAAAGCCAGACATGCCGATATTC |
| miR-122-5p primers | ID: #CD201-0006 (Tiangen Co. Ltd) |
| miR-185-5p primers | ID: #CD201-0393 (Tiangen Co. Ltd) |
| U6 primers | ID: #CD201-0145 (Tiangen Co. Ltd) |
| UCA1 siRNA: | AAGGAACATCTCACCAATTTC |
|  | AACCACCTTTAACTGTAGCTT |
|  | AATGGACAACAGTACACGCAT |
|  | TTATATCTTGAGACCCTATCC |
| Control siRNA: | AATTCTCCGAACGTGTCACGT |
| miR-122-5p | UGGAGUGUGACAAUGGUGUUUG |
