# Supplementary figures and images for: IMP1 regulates UCA1-mediated cell invasion through facilitating UCA1 decay and decreasing the sponge effect of UCA1 for miR-122-5p
Source: Breast Cancer Res. 2018 Apr 18;20:32. doi: 10.1186/s13058-018-0959-1 (PMC5907460; doi:10.1186/s13058-018-0959-1)

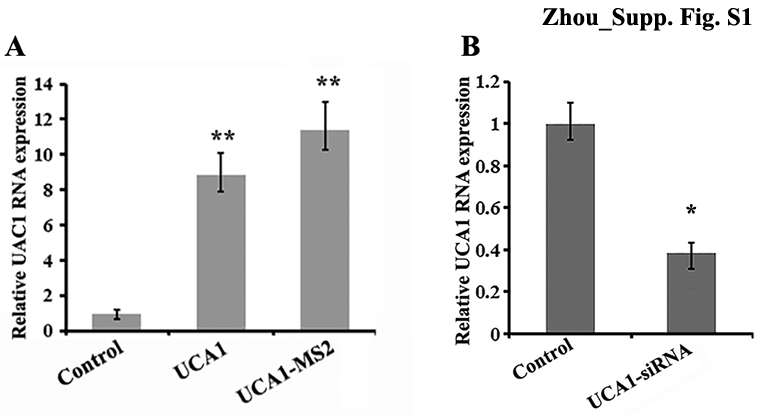

Supplement: Supplementary file 3 — Figure S1. UCA1 expression in MDA-MB-231 stable cell lines. (A) MDA231 cell lines stably expressing UCA1 or UCA1-MS2 were established. RT-qPCR analysis was performed to verify UCA1 expression. UCA1 levels were normalized to GAPDH mRNA from three independent experiments: **P < 0.01. (B) UCA1 knockdown MDA231 stable cell line was established by a lentivirus expressing UCA1-shRNA. RT-qPCR assays indicated that expression of UCA1 was knocked down by about 60%. Assays were normalized to GAPDH mRNA from three independent experiment:. **P < 0.01. (TIFF 336 kb) [file 13058_2018_959_MOESM3_ESM.tif]

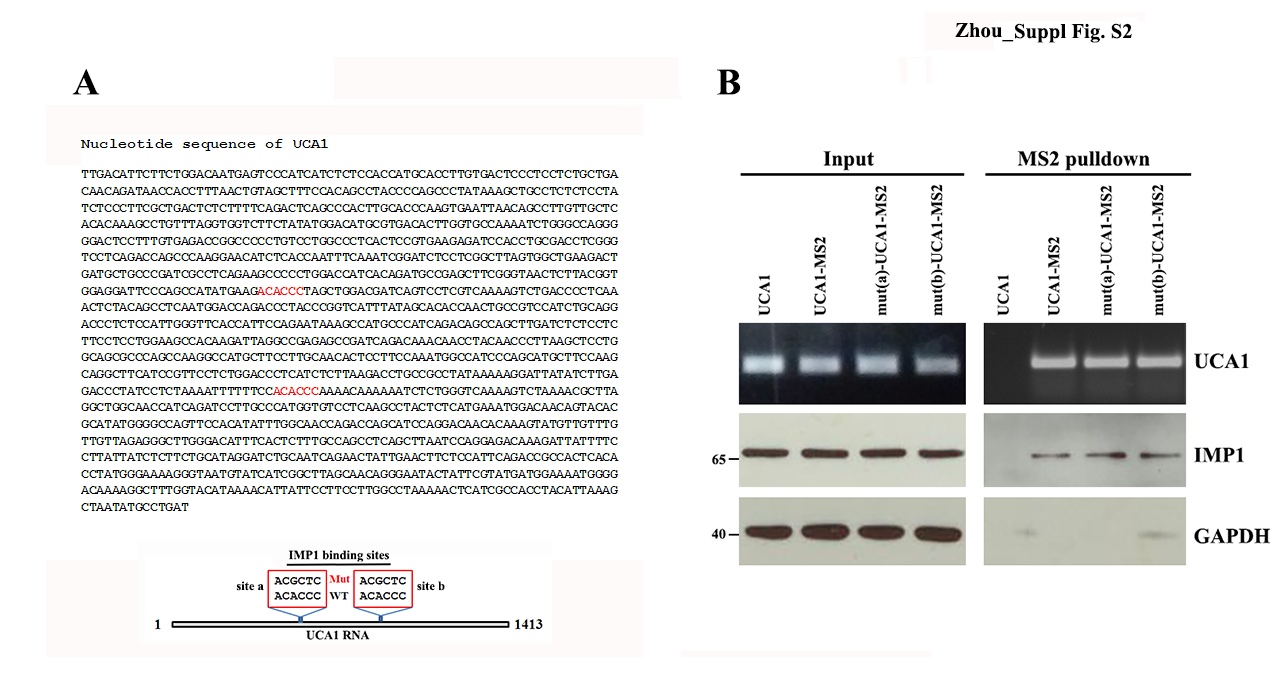

Supplement: Supplementary file 4 — Figure S2. UCA1 sequence and mutagenesis. The putative motifs for IMP1 binding in UCA1 are indicated in red. Mutations of the putative binding sites are shown below the UCA1 sequences. (TIFF 2587 kb) [file 13058_2018_959_MOESM4_ESM.tif]

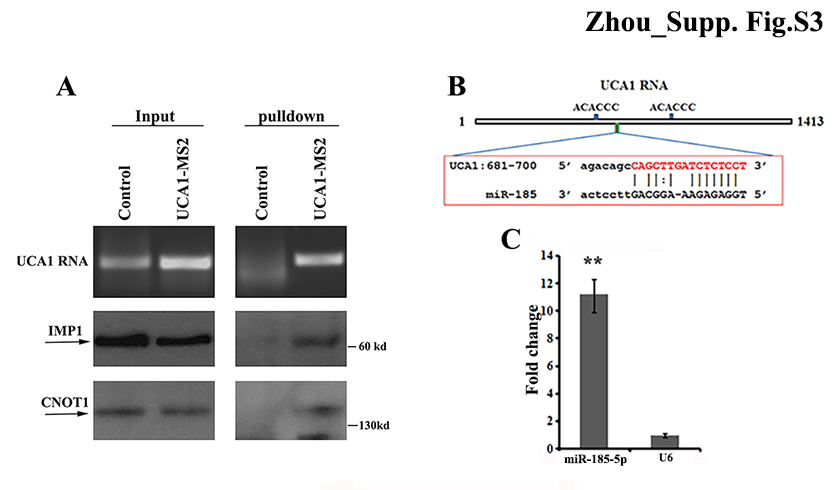

Supplement: Supplementary file 5 — Figure S3. UCA1 is associated with IMP1 and CNOT1 and with miR-185-5p. (A) Vectors expressing UCA1 or UCA1-MS2 were transiently transfected into MDA231/IMP1-GFP cells. Pulldown assays were performed to analyze the association of IMP1 and CNOT1 with UCA1-MS2. Representative images indicate that both IMP1 and CNOT1 co-precipitated with UCA1. Control: cells transfected with MS2-untagged UCA1. (B) Putative binding site of UCA1 for miR-185-5p. (C) Interaction of miR-185-5p with UCA1-MS2 was examined in the pulldown material. Relative levels of miR-185-5p in the precipitates were statistically analyzed as means ± SD from three independent experiments: **P < 0.01 as determined by Student’s t test. (TIFF 1227 kb) [file 13058_2018_959_MOESM5_ESM.tif]

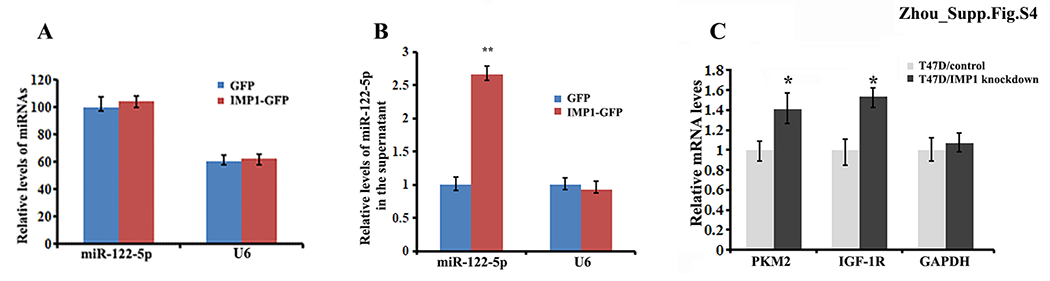

Supplement: Supplementary file 7 — Figure S4. IMP1 knockdown increases the expression of miR-122-5p target mRNAs. (A) Cellular levels of miR-122-5p are not affected by IMP1-GFP expression. (B) After MS2 pulldown experiments, levels of miR-122-5p in the supernatants were analyzed by qPT-PCR. Levels of miR-122-5p were normalized to GAPDH mRNA from three independent experiments: **P < 0.01 as determined by Student’s t test. (C) RT-qPCR was applied to measure the levels of PKM2 and IGF-1R mRNAs in IMP1 knockdown T47D cells. Levels of the mRNAs were normalized to GAPDH mRNA from three independent experiments: *P < 0.05 as determined by Student’s t test. (TIFF 884 kb) [file 13058_2018_959_MOESM7_ESM.tif]

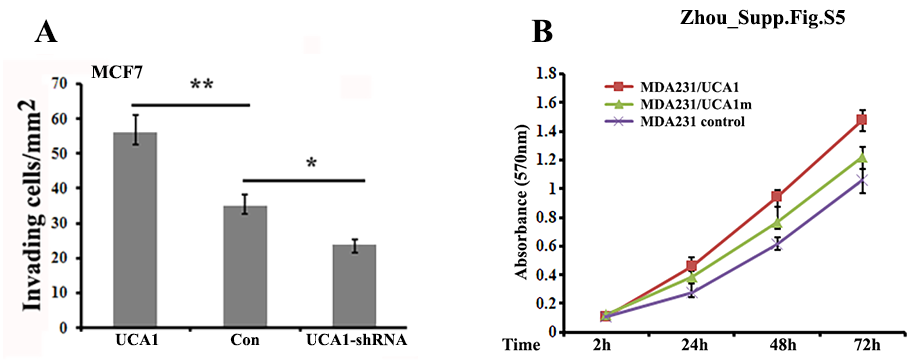

Supplement: Supplementary file 8 — Figure S5. Effect of UCA1 on the invasive abilities of MCF7 cells. Histograms show the effect of UCA1 on the invasive abilities of MCF7 cells. Values represent the means ± SD from three independent experiments; **P < 0.01, *P < 0.05 as determined by one-way ANOVA followed by Tukey’s multiple comparison tests. (TIFF 992 kb) [file 13058_2018_959_MOESM8_ESM.tif]

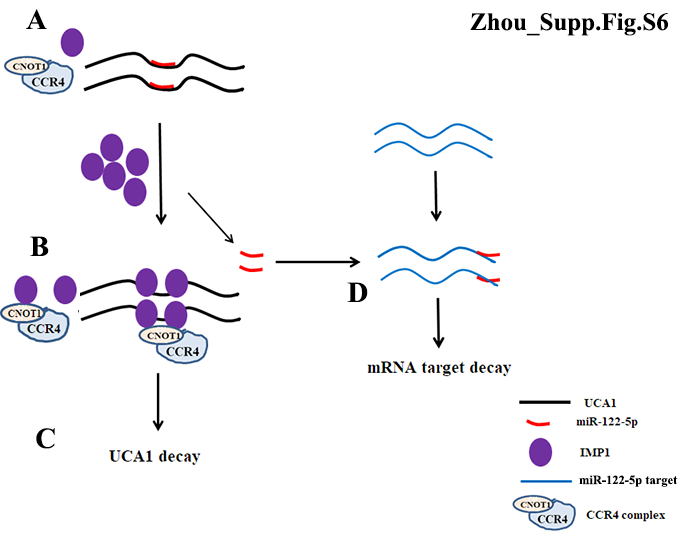

Supplement: Supplementary file 9 — Figure S6. A proposed model of IMP1 to regulate the sponge effect of UCA1 for miR-122-5p. (A) UCA1 sponges miR-122-5p, reducing miR-122-5p interaction with target mRNA. (B) Increasing IMP1 expression allows IMP1 to bind to UCA1 and to release miR-122-5p from UCA1. This increases the availability of miR-122-5p to interact with target mRNA. (C) Binding to target mRNA allows miR-122-5p to assert its posttranscriptional function. (D) IMP1 binds to UCA1 and recruits it to the CCR4-NOT1 complex, initiating UCA1 decay process. (TIFF 1116 kb) [file 13058_2018_959_MOESM9_ESM.tif]
