# Supplementary material for: IMP1 regulates UCA1-mediated cell invasion through facilitating UCA1 decay and decreasing the sponge effect of UCA1 for miR-122-5p
Source: Breast Cancer Res. 2018 Apr 18;20:32. doi: 10.1186/s13058-018-0959-1 (PMC5907460; doi:10.1186/s13058-018-0959-1)
Supplement: Supplementary file 6 — Table S2. miRNAs associated with UCA1. (DOCX 13 kb) [file 13058_2018_959_MOESM6_ESM.docx]

Table S2

| miRNA_ID | Fold change  (UCA1-MS2/MS2) |
| --- | --- |
| hsa-miR-122-5p | 11.9417 |
| hsa-miR-3591-3p | 10.9178 |
| hsa-miR-10b-5p | 9.6692 |
| hsa-miR-200a-3p | 7.6548 |
| hsa-miR-155-5p | 7.1809 |
| hsa-miR-185-5p | 7.0985 |
| hsa-miR-194-5p | 6.8183 |
| hsa-miR-26b-5p | 6.8183 |
| hsa-miR-31-5p | 6.5959 |
| hsa-miR-365b-5p | 6.5959 |
| hsa-miR-135a-5p | 6.5959 |
| hsa-miR-1-3p | 6.3329 |
| hsa-miR-7641 | 3.7528 |
| hsa-miR-4709-5p | 2.539 |
| hsa-miR-20a-5p | 2.1509 |
